# Evaluating Pancreatic and Biliary Neoplasms with Small Biopsy-Based Next Generation Sequencing (NGS): Doing More with Less

**DOI:** 10.3390/cancers14020397

**Published:** 2022-01-13

**Authors:** Ilias P. Nikas, Giannis Mountzios, Guy I. Sydney, Kalliopi J. Ioakim, Jae-Kyung Won, Panagiotis Papageorgis

**Affiliations:** 1 School of Medicine, European University Cyprus, Nicosia 2404, Cyprus; gsydney36@siumed.edu (G.I.S.); kjioakim@outlook.com (K.J.I.); 2 Fourth Department of Medical Oncology and Clinical Trials Unit, Henry Dunant Hospital Center, 11526 Athens, Greece; gmountzios@gmail.com; 3 Department of Internal Medicine, Southern Illinois University School of Medicine, Springfield, IL 62702, USA; 4 Department of Internal Medicine, Limassol General Hospital, Limassol 4131, Cyprus; 5 Department of Pathology, Seoul National University Hospital and College of Medicine, Seoul 03080, Korea; jkwon@snuh.org; 6 Tumor Microenvironment, Metastasis and Experimental Therapeutics Laboratory, Basic and Translational Cancer Research Center, Department of Life Sciences, European University Cyprus, Nicosia 2404, Cyprus; p.papageorgis@euc.ac.cy

**Keywords:** pancreatic neoplasms, endoscopic-ultrasound-guided fine needle aspiration (EUS-FNA), pancreatic juice, circulating tumor DNA (ctDNA), pancreatic cyst, biomarkers, tumor, molecular targeted therapy, biopsy, needle, liquid biopsy, pathology, molecular

## Abstract

**Simple Summary:**

Pancreatic cancer and cholangiocarcinoma are aggressive diseases mostly diagnosed at an advanced and inoperable stage. This review presents the value of next-generation sequencing (NGS) when performed on small biopsies—including fine-needle aspiration/biopsy samples, brushings, pancreatic juice and bile, and also blood—in the field of pancreatobiliary neoplasia. NGS could guide physicians while evaluating pancreatic solid and cystic lesions or suspicious biliary strictures, performing surveillance in high-risk individuals, or monitoring the disease and assessing prognosis in already diagnosed cancer patients. Evidence suggests that NGS performed on small biopsies is a robust tool for the diagnosis and pre-operative risk stratification of pancreatic and biliary lesions, whereas it also carries significant prognostic and therapeutic value. However, effective standardization of the pre-analytical and analytical assay parameters used for each clinical scenario is needed to fully implement NGS into routine practice and provide more personalized management in patients with suspected or established pancreatobiliary neoplasia.

**Abstract:**

Pancreatic cancer and cholangiocarcinoma are lethal diseases mainly diagnosed at an inoperable stage. As pancreatobiliary surgical specimens are often unavailable for further molecular testing, this review aimed to highlight the diagnostic, prognostic, and therapeutic impact of next-generation sequencing (NGS) performed on distinct small biopsies, including endoscopic ultrasound fine-needle aspirations and biopsies of pancreatic solid and cystic lesions, biliary duct brushings, and also “liquid biopsies” such as the pancreatic juice, bile, and blood. NGS could clarify indeterminate pancreatic lesions or biliary strictures, for instance by identifying TP53 or SMAD4 mutations indicating high-grade dysplasia or cancer. It could also stratify pancreatic cystic lesions, by distinguishing mucinous from non-mucinous cysts and identifying high-risk cysts that should be excised in surgically fit patients, whereas the combination of cytology, elevated cystic CEA levels and NGS could improve the overall diagnostic accuracy. When NGS is performed on the pancreatic juice, it could stratify high-risk patients under surveillance. On the plasma, it could dynamically monitor the disease course and response to therapy. Notably, the circulating tumor DNA (ctDNA) levels have been associated with staging, grading, and survival. Lastly, NGS has shown potential in identifying potentially actionable molecular alterations. In conclusion, NGS applied on small biopsies could carry significant diagnostic, prognostic, and therapeutic value.

## 1. Introduction

Pancreatic cancer is a lethal malignancy with a very low 5-year survival rate [1,2]. Notably, it causes almost the same number of deaths and new cases, according to the GLOBOCAN 2020 estimates [3]. Pancreatic cancer is mainly detected at a locally advanced or metastatic stage; thus, most patients are unfit for surgery at diagnosis, yet a few become eligible after neoadjuvant therapy (NAT) [4,5]. Chemotherapy is the treatment of choice, and common schemes include FOLFIRINOX (folinic acid, fluorouracil, irinotecan, oxaliplatin) or gemcitabine plus nab-paclitaxel [6,7]. Pancreatic adenocarcinoma (PDAC) is the most prevalent histologic type of pancreatic cancer, whereas PDAC precursors include pancreatic intraepithelial neoplasia (PanIN) with low-grade dysplasia (LGD) or high-grade dysplasia (HGD), and also intraductal papillary mucinous neoplasm (IMPN) and mucinous cystic neoplasm (MCN) with LGD or HGD [5,8]. PanINs are microscopic lesions giving rise to most PDACs, whereas IPMNs and MCNs are macroscopic lesions [2]. Aside from PDAC, other examples of pancreatic malignancies include acinar cell carcinoma, solid pseudopapillary neoplasm (SPN), and the pancreatic neuroendocrine neoplasms (NENs), which comprise neuroendocrine tumors (NETs) and carcinomas (NECs) [9,10]. Whereas distinguishing a solid neoplasm (e.g., PDAC, another pancreatic malignancy or a metastasis) from pancreatitis is the main differential diagnosis in the evaluation of solid pancreatic lesions [11,12], cystic lesions encompass various pathologies including non-neoplastic (e.g., pseudocyst), neoplastic benign, such as serous cystadenoma (SCA), neoplastic mucinous carrying malignant potential (e.g., IPMN or MCN), and malignant entities (e.g., IPMN or MCN with associated invasive carcinoma) [12,13,14,15]. Cholangiocarcinoma, a cancer arising in the biliary tract, is a rare malignancy mainly diagnosed at an advanced and inoperable stage, resulting in dismal prognosis [16].

At the molecular level, mutations at the following genes are most often identified in PDACs: KRAS, CDKN2A/p16, TP53, and SMAD4/DPC4 [2,6,17]. According to the PDAC progression model published some years ago, KRAS mutations are detected early, whereas the inactivating TP53 and DPC4 mutations occur later during the PDAC carcinogenesis [18,19,20]. Of interest, although KRAS mutations could be detected in low- or high-grade PanINs and IPMNs/MCNs, they could also be found in non-neoplastic disorders such as in chronic pancreatitis. In contrast, TP53 and SMAD4 alterations generally indicate the presence of HGD or cancer [21,22,23,24,25,26,27]. While evaluating a pancreatic cyst, finding a KRAS mutation favors a mucinous (e.g., IPMN or MCN) vs. a non-mucinous cyst (e.g., SCA or pseudocyst); moreover, an additional GNAS mutation indicates the presence of IPMN rather than MCN [28,29,30].

Next-generation sequencing (NGS) is an evolving modality that can simultaneously detect and quantify multiple genomic or transcriptomic targets in a single run and with a high analytical sensitivity [31,32]. In the era of precision and personalized medicine, NGS testing is often used in several clinical oncology applications [33]. Diverse sample types could be utilized, including limited tissue and cytologic samples, in addition to “liquid biopsies” such as blood, urine, pleural, and cerebrospinal fluids [34,35,36,37,38,39].

Sampling of pancreatic lesions is most often performed with endoscopic ultrasound fine-needle aspiration (EUS-FNA), fine-needle biopsy (FNB), or a combination of the two [40,41]. However, due to various reasons such as inadequate material or low cellularity, such sampling could result in non-diagnostic or indeterminate interpretations [42]. Pancreatic juice, collected in the duodenum following secretin stimulation, has been studied in high-risk individuals undergoing surveillance [23]. Blood-based liquid biopsies could be used to assess prognosis, select for targeted therapy, and dynamically monitor cancer progression, most often at an advanced stage; these are mainly composed of circulating tumor cells (CTCs), cell-free DNA (cfDNA—a component of which is the circulating tumor DNA (ctDNA), and exosomes [43]. Assessing biliary strictures is a challenging task, whereas tissue sampling includes brush cytology and/or biopsy [44]. Notably, as most PDACs and cholangiocarcinomas are inoperable at diagnosis, the surgical specimen is mainly unavailable for further molecular testing. Additionally, chemotherapy is often ineffective with short median survival [4,5,7,16]. Thus, checking for potentially targetable molecular alterations in FNAs, FNBs, blood, or any other “small biopsy” would be valuable for PDAC or cholangiocarcinoma patients [16,45,46,47].

This review aims to evaluate the diagnostic, prognostic, and therapeutic impact of the small biopsy-based NGS while assessing pancreatic and biliary lesions. We initially describe the impact of NGS performed on pancreatic FNAs, FNBs, and pancreatic juice, then focus on its value while evaluating biliary tract strictures (brushings, forceps biopsies, and bile) as well as blood-based liquid biopsies, and close with the discussion of our findings and conclusions.

## 2. The Role of NGS Performed on Pancreatic Small Biopsies

The summary of the published studies reporting on the role of small biopsy-based NGS in the evaluation and management of pancreatic lesions is shown in Table 1. Most studies highlighted its value in diagnosis (e.g., indeterminate case clarification or pre-operative stratification) or targeted therapy selection.

### 2.1. Most Common Mutations Detected in PDACs

NGS was performed on distinct small biopsy types, including EUS-FNAs or FNBs, brushings, and pancreatic juice, whereas several NGS panels were utilized. The initial material used for nucleic acid extraction was either fresh, directly collected for further NGS testing [25,55,61], frozen [23,57], also derived from formalin-fixed, paraffin-embedded tissue or cell blocks [70,71,85], residual liquid-based cytology (LBC) samples [58,62], or cytology slide scraping [59,60]. Mutations in the KRAS, TP53, CDKN2A, and SMAD4 genes were the most common ones detected in the PDAC patients tested [22,60,72,77]. Although KRAS mutations, as an early carcinogenic step, were also found in non-malignant cases, TP53 and SMAD4 alterations indicated HGD or carcinoma, triaging surgically fit patients for surgery [22,23,24,25,26,27].

### 2.2. Preoperative Evaluation of Pancreatic Cysts

A few of the published studies aimed to unravel the value of NGS in the preoperative evaluation of pancreatic cysts, in order to reduce unnecessary surgical procedures. This challenging task emerges more often in recent years, as more incidental cysts are detected, following the prevalent use of enhanced imaging technology [86]. To manage pancreatic cysts effectively, physicians should generally decipher if: (a) the cysts are mucinous or non-mucinous (the latter can be either non-neoplastic or benign with minimal malignant potential which can safely be managed conservatively), and (b) there is presence of at least HGD within the cysts; these would be classified as high-risk cysts, which are triaged for surgery [42]. In accordance with the literature, this review also found that the presence of KRAS mutations supported the diagnosis of a mucinous (IPMN or MCN) over a non-mucinous cyst (e.g., pseudocyst or SCA), whereas GNAS mutations favored IPMN over MCN [25,29,65,66,76,79]. NGS enhanced the diagnostic accuracy of EUS-FNA cytology to detect neoplastic mucinous cysts and differentiate them from the non-mucinous ones [49]. Notably, evidence indicated that NGS was more sensitive than the cytologic examination or elevated CEA cystic fluid levels (≥192 ng/mL), which are the two modalities traditionally used to evaluate pancreatic cysts [25,48,52,68]. For instance, Ren et al. showed the combination of cytologic examination, elevated CEA cystic fluid levels, and NGS reached a sensitivity of 94.1% and a specificity of 100% for the detection of neoplastic mucinous cysts [48]. Apart from discriminating between mucinous and non-mucinous pancreatic cysts, NGS was able to robustly identify high-risk cysts. A few studies indicated that specific mutations detected with NGS were associated with cystic neoplasms exhibiting HGD or invasion [25,76,87]. Rosenbaum et al. examined 113 pancreatic cystic fluid lesions from 105 patients and reported that SMAD4, TP53, CDKN2A, or NOTCH1 mutations indicated the presence of IPMN with high-grade dysplasia or cancer. Of interest, NGS combined with cytology improved the overall diagnostic accuracy to detect IPMNs and identify the high-risk IPMNs [25]. Similarly, Jones et al. also found that the presence of SMAD4, TP53, or CDKN2A alterations, discovered with NGS, indicate IPMNs with high-grade dysplasia or invasion [76].

### 2.3. Evaluation of High-Risk Patients under Surveillance with Pancreatic Juice-Based NGS

Likewise, some teams utilized pancreatic juice-based NGS to recognize HGD or cancer while evaluating solid or cystic pancreatic lesions. For instance, TP53 or multiple KRAS mutations were associated with invasive IPMNs [24,67]. Furthermore, Suenaga et al. tested the pancreatic juice from a mixture of pancreatic cancer and precursors (with both LGD and HGD) under surveillance, in addition to normal controls. They found that patients with HGD or cancer exhibited higher numbers and concentration of mutations other than KRAS/GNAS (also a higher overall mutation concentration) in their pancreatic juice. Mutations in TP53 and/or SMAD4 or a high SMAD4/TP53 mutation score were associated with HGD or cancer, whereas none of them were detected in the controls. Thus, NGS facilitated the stratification of high-risk patients under pancreatic surveillance, by identifying the patients harboring at least HGD [23]. Yu et al. applied pancreatic juice-based NGS in a cohort of 115 pancreatic solid and cystic lesions (34 PDACs, 57 IPMNs, and 24 non-neoplastic controls). They reported that PDAC patients showed higher mutation concentrations than IPMNs or controls. Although TP53 and SMAD4 mutations were associated with PDACs, they were also detected in 15/57 and 1/47 of IPMNs, respectively, but in none of the controls. Notably, two high-risk patients of the cohort under surveillance showed TP53/SMAD4 mutations more than a year before their cancer diagnosis [26].

### 2.4. Identification of Potentially Actionable Mutations in PDAC Patients

Apart from its use in diagnosis and preoperative risk stratification of pancreatic solid and cystic lesions, small biopsy-based NGS also showed potential in identifying potentially actionable alterations in PDAC patients. Takano et al. found such alterations in 22.4% of the cases tested [50], whereas Elhanafi et al. identified actionable mutations in the BRAF, MET, ERBB2, ARID1A, and BRCA1 genes in a few of the PDACs tested [70]. Lastly, Valero et al. reported at least one mutation in 17/19 of PDAC patients of their cohort, whereas KRAS, TP53, SMAD4, and ARID1A mutations were the ones most commonly detected. Notably, actionable alterations (e.g., in ATM or mTOR genes) were also found in some samples [46].

### 2.5. Evaluation of Neoplasms Other Than PDAC and Its Precursors

Whereas most studies focused on PDAC and its precursors, small biopsy-based NGS was also used to evaluate the molecular profile of other pancreatic neoplasms, pointing to a specific diagnosis or providing additional prognostic and therapeutic information. Gleeson et al. tested 90 primary and 32 metastatic PanNETs from the liver and reported that the former most often harbored MEN1, DAXX, ATRX, and TSC2 mutations. In addition, they found that alterations in TSC2, KRAS, and TP53 genes were associated with poor prognosis, whereas they also identified potentially actionable alterations in some members of the mTOR pathway (PTEN, TSC2, and PIK3CA) in 10% of primary and 12.5% metastatic NETs tested [74]. Whereas KRAS mutations were often in PDACs and IPMNs, they were not detected in the PanNET cases tested in two studies [58,83]. VHL mutations indicated a diagnosis of SCA in some studies. Of interest, Vestrup Rift et al. found that although mutations in KRAS and GNAS genes were the most common ones found in IPMNs, they were not detected in the three SCAs tested [66,68,76]. Furthermore, the presence of a CTNNB1 mutation indicated SPN; Kubota et al. found a CTNNB1 mutation in all seven SPNs, yet in just 1/11 NETs and in none of the PDACs, acinar cell carcinomas and pancreatitis cases of their cohort [81].

### 2.6. NGS Performed on FNA vs. Tissue Biopsy Samples

Evidence has shown that FNA-based NGS was highly concordant with its matched tissue-based molecular analysis, where it often revealed additional alterations, modifying the management plan of the patients [54,61,73,75,85]. In addition, it exhibited superior sensitivity than PCR or Sanger sequencing [83]. Rapid on-site evaluation (ROSE), besides improving the diagnostic accuracy of EUS-FNA, facilitated the acquisition of material for subsequent NGS testing, sparing the patients from additional invasive procedures [60]. Of interest, FNB was more likely to result in adequate material for subsequent NGS testing than FNA (OR: 4.95; 95% CI: 1.11–22.05; *p* = 0.04) [70], whereas larger gauge biopsy needles were more likely to result in successful NGS findings [64].

## 3. The Role of NGS Performed on Biliary Small Biopsies

The summary of the published studies reporting on the role of small biopsy-based NGS in the evaluation and management of suspicious biliary strictures is shown in Table 2.

NGS was performed on biliary tract brushings, forceps biopsies, and bile, whereas the authors utilized LBC samples or material directly collected for molecular evaluation [78,88,89,90,91,92]. Mutations in the KRAS, TP53, SMAD4, and CDKN2A genes were the most common ones detected [78]. Diagnostic accuracy was found to be relatively high. Rosenbaum et al. examined 96 strictures from 88 patients and reported that NGS exhibited higher sensitivity than cytology, whereas the presence of TP53, SMAD4, and CDKN2A mutations was 100% specific to detect HGD or cancer [90]. Furthermore, Singhi et al. examined 346 benign, premalignant, and malignant strictures from 252 patients and reported a sensitivity of 73% and specificity of 100% for malignancy. NGS exhibited an enhanced performance compared with the CA19-9 serum levels or the pathologic evaluation (performed on biliary brushings, biopsies, or both), whereas it also improved the overall diagnostic accuracy when combined with the pathologic evaluation. Notably, it also revealed potentially actionable alterations, such as the ERBB2 amplification in a few patients [92]. Two other studies additionally reported an improvement in the diagnostic accuracy, when the results of NGS were combined with the cytomorphologic evaluation [78,91]. Furthermore, NGS was found to be more sensitive, specific, and accurate than FISH, an already established method used to triage indeterminate biliary tract specimens [78].

Two research groups evaluated the potential of bile-based NGS in the evaluation of suspicious biliary strictures. NGS was more sensitive to detect malignancy, compared with the initial pathomorphological evaluation, performed either with FNA or FNB [88]. Notably, results were highly concordant with the molecular analysis performed in the matched tissue specimens, as 96.2% of the alterations present in the tissues were detected with bile-based NGS [89].

## 4. The Role of NGS Performed on Blood-Based Liquid Biopsies

The published evidence concerning the role of blood liquid biopsy-based NGS in the evaluation of pancreatic neoplasms is summarized in Table 3. Most studies highlighted its value in monitoring patients already diagnosed with locally advanced or metastatic PDAC, besides assessing prognosis or selecting the most appropriate targeted therapy in this clinical setting.

### 4.1. Monitoring Disease Course and Response to Therapy in PDAC Patients

Most studies used plasma cfDNA and targeted gene panels for NGS analysis, whereas the blood collection point ranged from treatment-naive patients (before chemotherapy or surgery), and also from patients during therapy and at disease progression. As it allows serial sampling, plasma-based liquid biopsy has shown great potential in the dynamic monitoring of the disease course and response to therapy of PDAC patients. Berger et al. performed NGS using the plasma cfDNA from 20 patients with metastatic PDAC and reported that their mutational landscape was often altered from baseline to the first, second, and third lines of treatment. Of interest, ctDNA quantity dropped from the baseline levels (before treatment initiation) during chemotherapy, whereas it surged during progression. In treatment-naive patients, the decrease in ctDNA quantity during therapy was associated with longer progression-free survival (PFS) [117]. Similarly, Park et al. tested 69 plasma cfDNA samples from 69 PDAC patients and found that the lowest ctDNA levels were associated with complete/partial disease response; thus, ctDNA levels were successful to monitor tumor burden, response to therapy, and disease progression [116]. Another study monitored 189 stage III and IV PDAC patients before, during chemotherapy, and together with each CT scan. They reported that ctDNA quantity was higher in stage IV than III PDACs, whereas higher ctDNA quantity was associated with disease progression and shorter overall survival (OS) at baseline, being a more significant prognostic marker than serum CA19-9 levels. CtDNA quantity changes during the sequential sampling predicted response to therapy in most of these patients [122]. In addition, Bachet et al. performed a randomized phase 2b trial enrolling 122 advanced PDAC patients, and showed that the presence of ctDNA at the first chemotherapy cycle was associated with shorter OS and PFS. Additionally, patients who responded to therapy exhibited negative or low ctDNA levels, whereas the ctDNA quantity alterations detected during sequential plasma sampling were associated with the overall response rate (ORR), OS, and PFS [101]. Notably, the presence of CTCs or ctDNA could be used to monitor PDAC patients receiving neoadjuvant therapy (NAT). A study by Yin et al. extracted the ctDNA and CTCs in a cohort composed of patients with pathologic complete response (pCR) after NAT. They found ctDNA in 7/16 and CTCs in 5/5 of the patients tested, suggesting their presence could indicate recurrence and worse survival [97].

### 4.2. Assessing Prognosis of PDAC Patients

As also displayed in the aforementioned studies, evidence suggests that the presence of plasma ctDNA and/or its levels are associated with the PDAC burden, staging, grading, and prognosis. Strijker et al. tested 77 plasma cfDNA samples from 58 metastatic PDAC patients and reported that ctDNA was most often found in patients with larger tumors and liver metastases. In addition, the ctDNA quantity was associated with 3D tumor volume (as measured by imaging), whereas it also predicted OS [108]. In another study, a higher tumor fraction was correlated with liver metastasis, shorter OS, and higher CA19-9 serum levels [100]. Pietratz et al. utilized plasma-based NGS in a cohort composed of resectable, locally advanced, and metastatic PDACs, and demonstrated that the presence of ctDNA was associated with tumor grade and stage (higher detection rates in high-grade and metastatic PDACs). Additionally, ctDNA presence and quantity were associated with shorter OS in advanced PDACs, whereas its absence conferred longer OS and DFS in resected PDACs [120].

### 4.3. Identifying Potentially Actionable Mutations in PDAC Patients

Besides its ability to monitor the disease course and response to therapy and its prognostic value, plasma-based NGS could also identify potentially targetable alterations in PDAC patients. In one study, actionable alterations were detected in 14/48 patients (e.g., in ALK, ATM, EGFR, and PIK3CA) [123], whereas in another one, such alterations (e.g., in BRCA1, EGFR, MET, BRAF, PIK3CA, and ERBB2) were also identified [104]. In the study by Li et al., two patients were successfully treated with immune checkpoint and PARP inhibitors (PARPi), based on the detection of MLH1 and BRCA1 mutations, respectively [103]. Lastly, Vidula et al. detected BRCA1/2 mutations in the plasma cfDNA samples tested, tailoring patients for treatment with PARPi therapy, whereas they also identified mechanisms of PAPRi resistance, such as BRCA1/2 reversion mutations [47].

### 4.4. NGS Performed on Blood-Based Liquid Biopsy vs. Tissue Biopsy Samples

Although blood-based NGS exhibits low concordance compared with tissue biopsy-based molecular analysis [112,118], it could identify additional alterations not detected in its paired biopsies, thus reflecting intratumoral heterogeneity more efficiently [119,127]. Notably, cfDNA collected during progression could also reveal new mutations, indicating tumor evolution [119].

## 5. Discussion

**Figure 1:**
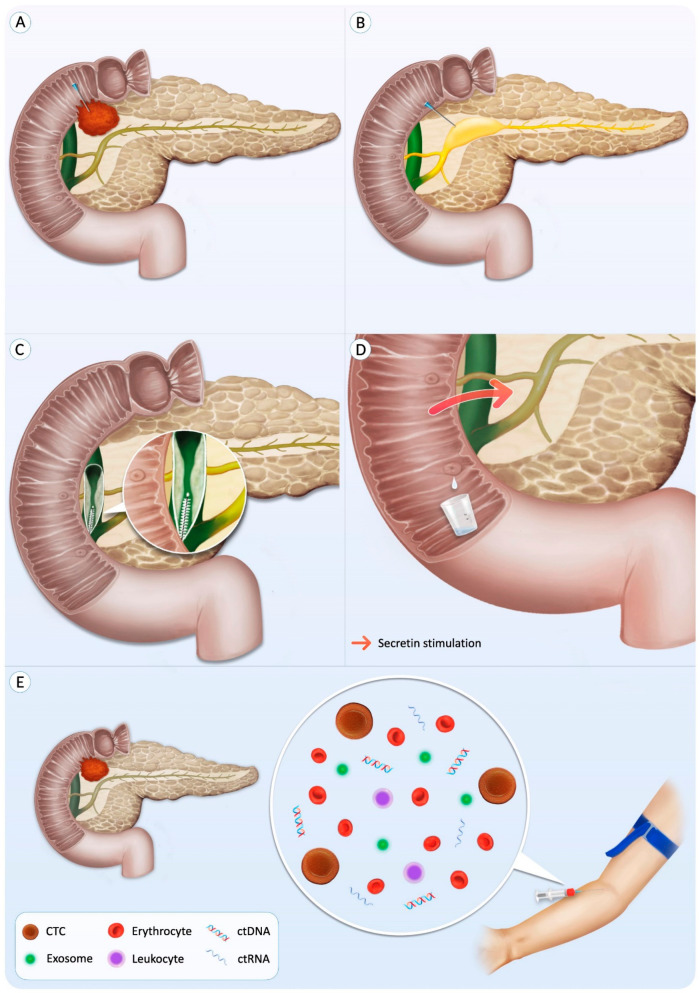
Next-generation sequencing can be performed on distinct small biopsies, including fine-needle aspiration of solid (**A**) and cystic (**B**) pancreatic masses, biliary brushings of biliary tract strictures (**C**), pancreatic juice collected from the duodenum (**D**), and blood-based liquid biopsy (**E**).

This review aimed to highlight the impact of small biopsy-based NGS in the evaluation of pancreatic and biliary neoplasms, guiding clinicians to provide personalized management for their patients. Evidence has shown that NGS could be applied with success in distinct small biopsies—including FNAs and FNBs of pancreatic solid and cystic lesions, pancreatic and biliary duct brushings, and liquid biopsies such as pancreatic juice, bile, and blood—providing answers to common clinical scenarios (Figure 1).

Firstly, NGS could help clarify indeterminate pancreatic or biliary cases by microscopy. According to the PDAC progression model, KRAS mutations are found early, whereas TP53 and DPC4 mutations occur later during the PDAC carcinogenesis [18,19]. Thus, although KRAS mutations could be detected in PanINs and IPMNs/MCNs of any grade or even in non-neoplastic cases, TP53 and SMAD4 alterations indicate the presence of HGD or cancer, triaging eligible patients for surgery [21,22,23,24,25,26,27]. Notably, Hosoda et al. selected 23 isolated HG-PanIN cases characterized by the absence of concurrent PDAC to perform molecular analysis. They reported that TP53 mutations were found in just 2/23 cases, whereas they did not find any non-synonymous SMAD4 alterations, suggesting that both mutations arise mostly at invasion [128]. Apart from its value assessing equivocal pancreatic lesions, NGS could be used in the evaluation and management of suspicious biliary strictures, as it has shown higher sensitivity than cytology or elevated serum CA19-9 levels to detect malignancy and an enhanced performance compared with FISH [78,88,89,90,91,92].

Furthermore, NGS could enhance the stratification of pancreatic cystic lesions—being an effective tool to distinguish mucinous from non-mucinous cysts—and identify high-risk cysts that should be excised in surgically fit patients [42]. Whereas the presence of KRAS mutations supports the diagnosis of a mucinous over a non-mucinous cyst, GNAS mutations favor the diagnosis of IPMN over MCN [25,29,65,66,76,79]. Evidence suggests that NGS has higher sensitivity than cytology or elevated cystic fluid CEA levels [25,48,52,68], whereas the combination of cytology, high CEA levels and NGS has shown the highest diagnostic accuracy to detect neoplastic mucinous cysts [48]. In addition, the presence of specific mutations—such as the ones in the SMAD4, TP53, CDKN2A, or NOTCH1 genes—have been linked with high-risk cysts [25,76,87]. To manage pancreatic cysts, clinicians most often use specific criteria described by organizations such as the International Association of Pancreatology (Fukuoka guidelines) [129] and the Americal Gastroenterological Association (AGA) [130]. For instance, the Fukuoka guidelines enlist distinct “high-risk stigmata” and “worrisome features”, which should be considered before deciding to surgically excise a pancreatic cystic lesion or recommend close follow-up. The presence of suspicious or positive cytology (microscopic features consistent with HGD or invasion) also triages eligible patients for surgery [131,132]; however, although pancreatic cyst cytology has a high specificity, its sensitivity is considered suboptimal [129]. Of interest, a recent meta-analysis on the Fukuoka and AGA guidelines found they both exhibited an inadequate diagnostic accuracy to distinguish between low- and high-risk pancreatic cysts [133]. Considering the findings presented in this review, the potential inclusion of NGS testing in these guidelines may enhance their diagnostic potential.

In the field of liquid biopsies, NGS has shown promising results when testing blood, pancreatic juice, and bile from patients with pancreatobiliary neoplasia. As blood-based liquid biopsy allows serial sampling (e.g., before chemotherapy or surgery, during therapy, and at disease progression), it has shown great potential in the dynamic monitoring of the PDAC disease course and response to therapy [97,101,117]. This is of great importance, especially when considering that traditional modalities used to monitor PDAC, such as the CA 19.9 serum levels and radiology, could exhibit suboptimal accuracy [134]. In addition, the presence of plasma ctDNA and/or its quantity have been associated with the PDAC burden, staging, grading, and prognosis [100,108,120], whereas they could often reflect heterogeneity more efficiently that tissue biopsy—revealing new mutations that indicate evolution—and potentially affect prognosis and response to therapy [119,127]. Of interest, a recent meta-analysis showed that the presence of ctDNA was associated with poor OS both at baseline and post-operatively (HR = 2.27; 95%CI (1.13–4.56) vs. HR = 3.66; 95%CI (1.45–9.28), respectively) in patients with resectable PDAC. Additionally, another meta-analysis compared the diagnostic accuracy of liquid with tissue-based molecular analysis, reporting that the former exhibited a pooled sensitivity and specificity of 70% and 86%, respectively, yet concordance was just 31.9% (as shown with a Venn diagram), when all mutations were considered [135].

Pancreatic juice could also be used to monitor PDAC patients after their surgery or individuals with high-risk to develop cancer. Suenaga et al. reported that mutations in TP53 and/or SMAD4 or a high SMAD4/TP53 mutation score were associated with HGD or cancer, whereas both were not detected in the control samples of the study. Thus, NGS facilitated the stratification of high-risk patients under pancreatic surveillance, through the identification of the patients harboring at least HGD [23].

As most PDAC patients are not eligible for surgery, another emerging application of NGS could be to identify potentially actionable alterations, such as in the BRAF, MET, ERBB2, ARID1A, BRCA1, ATM and mTOR genes [46,70]. This could even be carried out at the level of plasma-based NGS. For instance, the detection of mutations in the MLH1 or BRCA genes could tailor patients for treatment with immune checkpoint and PARP inhibitors, respectively [47,103]. Such findings could shift the direction of PDAC management away from the “one size fits all” chemotherapy approach towards precision oncology, as PDAC is not a single disease, albeit exhibiting molecular heterogeneity [136]. In addition, according to the alterations detected with NGS, patients could be selected for the most suitable clinical trials [137].

Lastly, small biopsy-based NGS could also identify alterations associated with other pancreatic lesions, pointing to a specific diagnosis or providing prognostic and therapeutic information. For instance, mutations in the MEN1, DAXX, and ATRX genes have been associated with PanNETs [74,138]. Additionally, VHL mutations indicate a diagnosis of SCA or a metastasis from a renal cell carcinoma [68,76,139,140], whereas CTNNB1 mutations a diagnosis of SPN [81].

To be successful, several pre-analytical and analytical parameters associated with any small-tissue based NGS need to be optimized. For instance, as PDAC generally contains abundant desmoplastic stroma, cellularity and tumor fraction could be low, negatively impacting the sensitivity of the reaction; thus, sample adequacy needs to be assessed before running an NGS reaction [141,142,143,144]. Some studies in our review showed that FNB was more likely than FNA to result in adequate material for subsequent NGS testing [64,70]. However, the application of ROSE could facilitate the acquisition of cytologic material to be further processed for NGS testing, sparing the patients from additional invasive procedures [60]. The findings of this review should be interpreted with caution, as there was significant variation in the clinical setting of the included studies (e.g., PDACs of various stages or various percentages of different disease entities), whereas some studies recruited small patient numbers to draw meaningful results. In addition, there was substantial heterogeneity in the preanalytical—for instance, regarding FNAs, NGS was performed on directly collected material, cytology slide scraping, supernatants from post-centrifuged or residual LBC samples—or analytical parameters of the NGS assays applied (e.g., diversity of gene panels or depth of coverage). Thus, it is imperative to validate the most robust assays for each pancreatobiliary small-biopsy application described in this review. Future research in the form large prospective studies or randomized clinical trials may strengthen the aforementioned findings.

## 6. Conclusions

Evidence suggests that NGS performed on small biopsies is a robust tool for the diagnosis and risk stratification of pancreatic and biliary lesions, whereas it also carries significant prognostic and therapeutic value. However, effective standardization of the pre-analytical and analytical assay parameters used for each clinical scenario is needed to fully implement NGS into routine practice.

## Figures and Tables

**Table 1:** The role of NGS performed on pancreatic small biopsies (EUS-FNA and FNB, brushings, and pancreatic juice): doing more with less.

First Author, Year	Small Biopsy Type Clinical Setting	NGS Strategy	Main Findings
Ren, 2021 [48]	EUS-FNA Pancreatic mucinous cystic lesions	48 gene panel	KRAS and/or GNAS mutations were detected in 59/68 cases tested; NGS was more sensitive to detect a neoplastic mucinous cyst than cytologic examination or elevated CEA cystic fluid levels, whereas their combination showed a sensitivity of 94.1% and a specificity of 100%; in 6/10 mucinous cysts without a KRAS mutation, a combination of BRAF and GNAS mutations were detected
Haeberle, 2021 [49]	EUS-FNA Pancreatic mucinous cystic lesions	50 gene panel	NGS enhanced the diagnostic accuracy of EUS-FNA cytology to detect neoplastic mucinous cysts
Takano, 2021 [50]	EUS-FNA/FNB PDACs	50 gene panel	Mutations in KRAS, TP53, SMAD4, and PTEN genes were the most common ones detected; 22.4% of the cases exhibited potentially targetable alterations
Perez, 2021 [51]	EUS-FNA Pancreatic cystic lesions	39 gene panel	KRAS and/or GNAS mutations were 83.3% sensitive and 60% specific to detect a neoplastic mucinous cyst
Schmitz, 2021 [52]	EUS-FNA Pancreatic mucinous cystic lesions	14 gene panel	KRAS or GNAS mutations were found in 43/47 patients tested; NGS exhibited higher sensitivity to detect a neoplastic mucinous cyst than cytology or elevated CEA levels
Kuratomi, 2021 [53]	Pancreatic juice IPMNs with and without invasion	miRNA sequencing	The miR-10a-5p was upregulated at a significant level in invasive, compared with noninvasive IPMNs
Sekita-Hatakeyama, 2021 [22]	FNA Pancreatic and periampullary lesions suspicious for malignancy	6 gene panel	Mutations in KRAS, TP53, CDKN2A, and SMAD4 genes were the most common ones detected; 18/33 PDACs were identified as carrying at least HGD (KRAS and CDKN2A/PIK3CA/TP53/SMAD4 mutations) with NGS performed on residual LBC specimens, whereas 10/11 benign cases showed no mutations
Habib, 2021 [54]	FNA; plasma cfDNA Lesions suspicious for PDAC	9 gene panel	FNA-based NGS identified 16/16 of the KRAS mutations found in their paired histological specimens, in contrast to 6/8 identified by the plasma-based molecular analysis; mutations in the KRAS and TP53 genes were the most common ones detected
Dupain, 2020 [55]	CT or EUS-FNA and EUS-FNB Pancreatic cancer metastases	87 gene panel	Among the metastatic tumors (e.g., from pancreas, breast, and colon) prospectively tested, FNA-based was highly concordant with the CNB-based NGS; potentially actionable alterations were also identified
De Biase, 2020 [56]	FNAs and direct fluid samples Solid and cystic pancreatic lesions	22 gene panel	KRAS p.G12V and p.G12D were the most common mutations detected in the 42 pancreatic lesions tested
Carrara, 2020 [57]	EUS-FNA and EUS-FNB PDACs	161 gene panel	In this clinical trial, NGS was successful in almost all samples tested and exhibited higher diagnostic yield (94%) than histology (91%) or cytology (88%); at least two mutations were found in the majority of PDAC cases, whereas KRAS mutations were the most common ones detected
Fulmer, 2020 [58]	EUS-FNA Solid and cystic pancreatic lesions	143 gene panel	DNA of high quality was retrieved from most samples; NGS revealed clinically significant mutations in 10/14 mucinous cysts (e.g., KRAS, GNAS, TP53 mutations) and 13/15 PDACs (KRAS mutations in 10 and TP53 in 9 samples), whereas it did not exhibit any mutation in the 4 PanNETs tested
Plougmann, 2020 [59]	EUS-FNA Solid pancreatic lesions	19 gene panel	Mutations in KRAS and TP53 were only detected in the malignant and indeterminate cases; NGS could aid in the stratification of imaging and cytology indeterminate cases
Ishisawa, 2020 [60]	EUS-FNA Pancreatic cancers	409 gene panel	In addition to improving the diagnostic accuracy of EUS-FNA, ROSE facilitated the acquisition of material for subsequent NGS testing, sparing patients from additional invasive procedures; mutations in KRAS, TP53, SMAD4, and CDKN2A genes were the most common ones detected
Laquiere, 2020 [61]	EUS-FNA Pancreatic cystic lesions	526 gene panel	Cystic fluid-based NGS was concordant with its paired post-surgical NGS testing in 15/17 matched samples, whereas it also identified additional molecular alterations; mutations in KRAS and GNAS genes were the most common ones detected
Paziewska, 2020 [27]	EUS-FNA Pancreatic cystic lesions	409 gene panel	Mutations were mostly found in the TP53, KRAS, PI3CA, and GNAS genes; except for IPMNs, MCNs, and malignant cysts, 13% of SCAs and 14% of pseudocysts also exhibited KRAS mutations
Yamaguchi, 2020 [62]	Pancreatic juice PDACs	28 gene panel	SMAD4, CDKN2A, and TP53 mutations were identified by performing NGS on residual LBC specimens
Sugimori, 2020 [63]	EUS-FNA PDACs	50 gene panel	NGS was performed in two PDACs and was concordant to digital PCR concerning the absence of KRAS G12/13 mutations; NGS additionally detected KRAS Q61K and TP53 mutations in one of the cases tested
Park JK, 2019 [64]	EUS-FNA and FNB PDACs	83 gene panel	Larger gauge needles were more likely to result in successful NGS results (OR = 2.19; 95% CI: 1.08 to 4.47; *p* = 0.031)
Volckmar, 2019 [65]	EUS-FNA Pancreatic cystic lesions	14 gene panel	Mutations were found in all tested IPMNs (*n* = 12), most often in the KRAS and GNAS genes, whereas none of the tested pseudocysts (*n* = 3) showed any KRAS/GNAS mutations; cellular fraction exhibited superior results than the liquid fraction molecular analysis
Vestrup Rift, 2019 [66]	EUS-FNB Pancreatic cystic lesions	50 gene panel	Mutations in KRAS and GNAS genes were the most common ones detected in IPMNs (11/19 and 13/19 cases, respectively), whereas the three SCAs tested did not show any mutations
Takano, 2019 [67]	Pancreatic juice IPMNs with and without invasive component	2 panels, targeting 50 and 6 genes	TP53 or multiple KRAS mutations were associated with invasive IPMN
Sakhdari, 2019 [68]	EUS-FNA Pancreatic cystic lesions	50 gene panel	NGS was more sensitive than cytology, whereas their combination improved the diagnostic sensitivity; KRAS and GNAS mutations were the ones most often detected, whereas SMAD4 and VHL mutations were found in PDACs and SCAs, respectively
Choi, 2019 [69]	Pancreatic juice PDACs	15 gene panel	Most pancreatic juice samples revealed KRAS mutations, even when these were not found in the resected primary tissue molecular analysis; six juice samples (29%) also revealed TP53 mutations, whereas the cases with a concurrent KRAS and TP53 mutational profile were concordant between the paired tissue and pancreatic juice molecular analysis
Elhanafi, 2019 [70]	EUS-FNA and FNB PDACs	47 gene panel	FNB was more likely to result in adequate material for subsequent NGS testing than FNA (OR = 4.95; 95% CI: 1.11–22.05; *p* = 0.04), especially in PDACs ≤ 3 cm or PDACs located in the head or neck of the pancreas; KRAS, TP53, and SMAD4 mutations were the most frequent mutations found, whereas actionable alterations (e.g., in BRAF, MET, ERBB2, ARID1A, and BRCA1 genes) were identified in several PDACs
Larson, 2018 [71]	EUS-FNA and FNB, forceps biopsies, percutaneous CNBs PDACs (also one ACC and one AAC)	324 gene panel	Adequacy for subsequent NGS analysis was significantly associated with larger-gauge needles and sampling of the metastatic lesions
Sibinga Mulder, 2018 [72]	EUS-FNA and brushings Pancreatic or periampullary lesions	50 gene panel	KRAS, TP53, SMAD4, and CDKN2A mutations were the ones most often detected; NGS exhibited high diagnostic accuracy and facilitated preoperative risk stratification, leading to management change in 10% of the patients
Suenaga, 2018 [23]	Pancreatic juice PDACs and precursors; non-neoplastic controls	12 gene panel	Patients with HGD or cancer showed higher number and concentration of mutations other than KRAS/GNAS (also higher overall mutation concentration) in their pancreatic juice; mutations in TP53 and/or SMAD4 or a high SMAD4/TP53 mutation score were associated with HGD or cancer, whereas they were not detected in the controls; NGS could facilitate the stratification of high-risk patients under pancreatic surveillance, by identifying patients harboring HGD or cancer
Takano, 2017 [24]	Pancreatic juice IPMNs	2 panels, targeting 50 and 6 genes	Mutations in the KRAS and GNAS genes were the most common ones detected, whereas TP53 mutations were associated with malignant IPMNs, both in the pancreatic juice and tumor resection specimens tested
Rosenbaum, 2017 [25]	EUS-FNA Pancreatic cystic lesions	39 gene panel	Mutations in the KRAS and GNAS genes supported the diagnosis of an IPMN over a non-mucinous cyst; additional non-KRAS/GNAS aberrations (SMAD4, TP53, CDKN2A, or NOTCH1 mutations) indicated the presence of IPMN with HGD or invasion; NGS improved the overall diagnostic accuracy when added to cytology for both the detection of mucinous vs. non-mucinous cysts and the presence of at least HGD (high-risk cysts)
Sibinga Mulder, 2017 [73]	EUS-FNA PDAC	50 gene panel	Mutations in KRAS, TP53, and CDKN2A were detected in both the EUS-FNA and matched tumor resection specimen tested (SMAD4 mutation was found only in the former); NGS modified the management plan of this patient
Yu, 2017 [26]	Pancreatic juice Pancreatic solid and cystic lesions, also non-neoplastic controls	9 gene panel	PDAC patients showed higher mutation concentrations than IPMNs or controls; mutations in the TP53 and SMAD4 genes were found most often in PDACs, whereas they were also detected in 15/57 and 1/57 of the IPMNs tested, respectively, albeit in none of the controls; KRAS mutations were also found in 10/24 of the controls; two high-risk patients under surveillance showed TP53 or SMAD4 mutations in the pancreatic juice-based molecular analysis, more than a year before their cancer diagnosis
Gleeson, 2017 [74]	EUS-FNA PanNETs (primary and liver metastases)	15 gene panel	Alterations in the MEN1, DAXX, ATRX, and TSC2 genes were the most common ones detected in primary PanNETs; TSC2, KRAS, and TP53 alterations were associated with poor prognosis; potentially actionable alterations in members of the mTOR pathway (PTEN, TSC2, and PIK3CA) were identified in 10% of the primary and 12.5% metastatic PanNETs tested
Gleeson, 2016 [75]	EUS-FNA PDACs, IPMNs with invasion, AACs	160 gene panel	Mutations in the KRAS, TP53, SMAD4, and GNAS genes were the most common ones detected; SMAD4 mutations were detected in nine patients, yet in none of the four AAC patients tested; FNA-based NGS was highly concordant with the matched tumor resection-based NGS analysis
Jones, 2016 [76]	EUS-FNA Pancreatic cystic lesions	39 gene panel	Mutations in the KRAS, GNAS, and CDKN2A genes were the most common ones detected; KRAS and GNAS mutations supported the diagnosis of IPMN, even when the CEA levels were low; additional non-KRAS/GNAS aberrations (SMAD4, TP53, or CDKN2A) indicated the presence of IPMN with HGD or cancer; VHL mutations supported the diagnosis of SCA
Valero, 2016 [46]	EUS-FNA Unresectable PDACs	409 gene panel	NGS revealed at least one mutation in 17/19 PDAC patients tested; mutations in KRAS, TP53, SMAD4, and ARID1A genes were the most common ones detected; actionable mutations (e.g., in the ATM or mTOR genes) were also detected in a few cases
Kameta, 2016 [77]	EUS-FNA Solid and cystic pancreatic lesions	50 gene panel	KRAS mutations were found in 26/27 PDAC albeit none of the non-PDAC cases; KRAS, TP53, CDKN2A, and SMAD4 mutations were the most common ones detected
Dudley, 2016 [78]	Main pancreatic and bile duct brushings Pancreatobiliary duct strictures	39 gene panel	Mutations in the KRAS, TP53, SMAD4, and CDKN2A genes were the most common ones detected; a KRAS mutation was also found in a non-neoplastic case (cholecystitis); NGS was more sensitive, specific, and accurate than FISH, whereas it improved the overall sensitivity and diagnostic accuracy when combined with cytology
Springer, 2015 [79]	EUS-FNA or direct collection from the resected tissue specimens Pancreatic cystic lesions	11 gene panel	KRAS and GNAS mutations were the most common ones found in IPMNs (78% and 58% of the cases, respectively); KRAS mutations were the most common ones found in MCNs (6/12 cases tested); CTNNB1 mutations were found in SPNs, whereas VHL mutations were found in SCAs
Wang, 2015 [80]	EUS-FNA Pancreatic cystic lesions	Non-coding RNA sequencing	miRNA expression profiling was used to distinguish low-grade from high-grade/malignant pancreatic cystic lesions; the latter showed enrichment of 13 and depletion of two miRNAs
Kubota, 2015 [81]	EUS-FNA Pancreatic solid and cystic lesions	WES (CTNNB1 gene)	A CTNNB1 mutation in exon 3 was found in all seven SPNs tested 1/11 NETs but none of the PDACs, ACC, or non-neoplastic cases tested displayed a CTNNB1 mutation
Di Marco, 2015 [82]	EUS-FNB PDACs	WTS	KRAS, TP53, SMAD4, and CDKNA mutations were the most common ones found in PDACs; ARID1A alterations were found in 6/16 of the PDACs tested, whereas PTEN inactivation was identified only in advanced PDACs
De Biase, 2014 [83]	EUS-FNA Pancreatic solid and cystic lesions	KRAS (exons 2 and 3)	KRAS mutations were found in most of the PDACs and IPMNs, but in none of the PanNET cases tested; NGS exhibited superior sensitivity than PCR or Sanger sequencing, whereas it maintained a high specificity; sensitivity was higher when cytology slide scraping of selected areas (rather than fresh aliquots) was used for NGS analysis
Amato, 2014 [84]	Direct cystic fluid collection from surgical specimens IPMNs	50 gene panel	GNAS, KRAS, and TP53 mutations were the most common ones found in PDACs
Takano, 2014 [29]	Pancreatic juice Pancreatic solid and cystic lesions	46 gene panel	GNAS mutations were found in 41.5% of the IPMNs tested; all PDAC cases with GNAS mutations had concurrent IPMN; GNAS mutations were associated with main duct IPMNs exhibiting dilatation ≥6 mm
Young, 2013 [85]	FNA PDACs (also one PanNET)	Exons of 287 and introns of 19 genes	Mutations in KRAS, TP53, CDKN2A/B, SMAD4, and PTEN were the most common ones found; FNA-based NGS was 100% concordant with its matched tissue-based NGS analysis for the aberrations discovered

Abbreviations: EUS-FNA, endoscopic ultrasound-guided fine-needle aspiration; EUS-FNB, endoscopic ultrasound-guided fine-needle biopsy; PDAC, pancreatic adenocarcinoma; IPMN, intraductal papillary mucinous neoplasm; MCN, mucinous cystic neoplasm; SCA, serous cystadenoma; SPN, solid pseudopapillary neoplasm; cfDNA, cell-free DNA; CNB, core needle biopsy; AAC, ampullary adenocarcinoma; ACC, acinar cell carcinoma; PanNET, pancreatic neuroendocrine tumor; WES, whole exome sequencing; WTS, whole transcriptome sequencing; NGS, next-generation sequencing; HGD, high-grade dysplasia; LBC, liquid-based cytology; ROSE, rapid on-site evaluation; FISH, fluorescence in in situ hybridization.

**Table 2:** The role of NGS performed on biliary small biopsies (bile duct brushings, forceps biopsy, and bile): doing more with less.

First Author, Year	Small Biopsy Type Clinical Setting	NGS Strategy	Main Findings
Arechederra, 2021 [88]	Bile Bile duct strictures	52 and 161 gene panels	NGS was more sensitive to detect malignancies compared with the initial pathologic evaluation (performed either with FNA or FNB);mutations in the KRAS, TP53, ERBB3, and GNAS genes were the most common ones detected
Driescher, 2020 [89]	Bile; plasma cfDNA Biliary obstruction (in PDAC and CCA patients)	50 gene panel	Bile-based NGS identified 96.2 % of the molecular alterations found in the paired histological specimens, in contrast to 31.6% identified by the plasma-based molecular analysis
Rosenbaum, 2020 [90]	Bile duct brushings (LBC samples) Bile duct strictures	39 gene panel	NGS exhibited higher sensitivity than cytology to diagnose HGD or cancer, whereas the presence of late mutations (TP53, SMAD4, CDKN2A) was 100% specific; KRAS/GNAS mutations were found in both benign and malignant strictures; selected cytomorphologic characteristics (anisonucleosis, nucleomegaly, coarse chromatin, and stripped nuclei) were associated with late rather than early (e.g., KRAS) mutations
Harbhajanka, 2020 [91]	Bile duct brushings Bile duct strictures	52 and 69 gene panels	NGS improved the overall diagnostic accuracy when combined with cytology; mutations were found in 93% of the malignant cases tested, most often in the KRAS and TP53 genes
Singhi, 2020 [92]	Bile duct brushings; forceps biopsies Bile duct strictures	28 gene panel	NGS exhibited a sensitivity of 73% and a specificity of 100% to detect malignancy, performing better than CA19-9 serum levels or the pathologic evaluation (conducted in biliary brushings, biopsies, or both); NGS also improved the overall diagnostic accuracy, when combined with the pathologic evaluation, both in the brushing and biopsy specimens; lastly, it revealed potentially actionable alterations (e.g., ERBB2 amplification) in 8% of the patients tested
Dudley, 2016 [78]	Bile and main pancreatic duct brushings Biliary and pancreatic duct strictures	39 gene panel	Mutations in the KRAS, TP53, SMAD4, and CDKN2A genes were the most common ones detected; a KRAS mutation was also found in a non-neoplastic case (cholecystitis); NGS was more sensitive, specific, and accurate than FISH, whereas it improved the overall sensitivity and diagnostic accuracy when added to cytology

Abbreviations: cfDNA, cell-free DNA; PDAC, pancreatic adenocarcinoma; CCA, cholangiocarcinoma; LBC, liquid-based cytology; NGS, next-generation sequencing; FNA, fine-needle aspiration; FNB, fine-needle biopsy; HGD, high-grade dysplasia; FISH, fluorescence in situ hybridization.

**Table 3:** The role of blood liquid biopsy-based NGS in the evaluation of pancreatobiliary lesions: doing more with less.

First Author, Year	Liquid Biopsy Yype Clinical Setting	NGS Strategy	Blood Collection Time Point	Main Findings
Affolter, 2021 [93]	Plasma cfDNA PDAC patients	118 gene panel	Before and after surgery	High ctDNA levels before surgery were significantly associated with poor survival
van der Sijde, 2021 [94]	Plasma cfDNA PDAC patients under chemotherapy	57 gene panel	Before and after the first chemotherapy cycle	TP53 mutations and the TP53 Pro72Arg germline variant were independent predictors of PDAC progression; this combination of genetic lesions was linked with poor OS
Botrus, 2021 [95]	Plasma cfDNA Patients with locally advanced or metastatic PDAC	54, 68, 70, 73, and 74 gene panels	Before and during treatment, also at disease progression	Mutations in TP53 and KRAS genes were the most common ones detected; almost half of the patients (48%) exhibited potentially targetable alterations, such as KRAS (G12C) and EGFR
Yu, 2020 [96]	DNA from CTCs Patients with stage IA, IIB, and IV PDAC, also one healthy control	scNGS; 3 gene panel	NA	Mutations (KRAS, 6/12 patients; TP53, 5/12 patients; and SMAD4, 3/12 patients) were found only in the patients with metastatic PDAC
Yin, 2020 [97]	Plasma cfDNA and CTCs PDAC patients with pCR after NAT	6 gene panel	At the time of surgery and during follow-up	ctDNA was detected in 7/16, whereas CTCs were found in 5/5 patients with pCR after NAT tested, suggesting recurrence and worse survival
Guo, 2020 [98]	Plasma cfDNA Patients with resectable PDAC	50 gene panel	Before surgery	NGS was highly concordant with digital PCR;KRAS mutations (especially the KRAS G12D) were associated with poor prognosis (shorter OS and RFS) and early distant metastasis
Metzenmacher, 2020 [99]	Plasma cfRNA Patients with stage III PDACs and healthy controls	Total RNA sequencing	Before treatment initiation	PDAC patients exhibited higher cfRNA quantity and POU6F2-AS expression than the controls
Vidula, 2020 [47]	Plasma cfDNA Patients with advanced PDAC	73 gene panel	NA	NGS detected germline, somatic, and reversion BRCA1/2 mutations, tailoring patients for treatment with PARPi therapy; NGS also identified mechanisms of PAPRi resistance (BRCA1/2 reversion mutations)
Wei, 2020 [100]	Plasma cfDNA Patients with locally advanced or metastatic PDAC	WGS	Before or following therapy; serial sampling (monitoring) for 14 patients	Higher tumor fraction was correlated with liver metastasis, shorter OS, and higher serum CA19-9 levels; CNAs were detected in almost half of the patients, especially in the ones with liver metastases, and were linked with favorable chemotherapy response; in the serial samples, tumor fraction estimated the tumor burden and response to treatment for most patients
Bachet, 2020 [101]	Plasma cfDNA Patients with advanced PDAC	22 gene panel	At first day of the first, second and third cycle of therapy	In this randomized phase 2b trial, presence of ctDNA at baseline was associated with shorter OS and PFS, also with response to eryaspase (patients who responded to therapy exhibited negative or low ctDNA levels); the ctDNA quantity alterations detected in the consecutive plasma samples were associated with ORR, OS, and PFS
Uesato, 2020 [102]	Plasma cfDNA Patients with metastatic PDAC	14 gene panel	Before or during therapy	Mutations in TP53 and KRAS were the most common ones found; ctDNA presence was associated with shorter OS and PFS, metastasis, tumor burden, and higher serum CA19-9 levels
Li, 2020 [103]	Plasma cfDNA PDAC patients	150 gene panel	NA	ctDNA was identified in almost 70% of the patients; mutations in KRAS, TP53 and CDKN2A were the most common ones found, whereas actionable alterations (e.g., in NTRK, BRCA1/2) were also identified; two patients were successfully treated with ICI or PARPi based on detected MLH1 and BRCA1 mutations, respectively
Zakka, 2020 [104]	Plasma cfDNA Patients with PanNET	73 gene panel	NA	Mutations in the TP53, KRAS, and APC genes were the most common ones found; potentially actionable alterations (e.g., in BRCA1 EGFR, MET, BRAF, PIK3CA, and ERBB2) were also identified
Yang, 2020 [105]	Plasma EV-derived RNA (NGS or qPCR); plasma cfDNA (digital or qPCR) Patients with PDAC, a non-PDAC pancreatic lesion, and healthy controls	miRNA sequencing	Before therapy (baseline)	Multi-analyte liquid biopsy (EV-derived mRNA/miRNA, cfDNA concentration, KRAS MAF, and CA19-9 levels) exhibited superior diagnostic accuracy to detect and stage PDACs than CA19-9 and imaging, respectively; this approach also spotted metastases missed by imaging at baseline, which were later discovered during surgery or follow-up imaging, exhibiting the potential to identify suitable surgical candidates
Macgregor-Das, 2020 [106]	Plasma cfDNA PDAC patients and healthy controls	Digital NGS: KRAS (codons 12, 13) and GNAS (codon 201)	Before surgery for resectable PDACs	Mutations in KRAS codon 12 were the most common ones detected; KRAS ctDNA combined with CA19-9 levels showed a diagnostic sensitivity of 66.7%; enzymatic pretreatment before digital NGS decreased the background errors of the assay, thus potential false positive results
Kumar, 2020 [107]	Exosomal RNA Patients with Stage III and IV PDACs, IPMNs, and healthy controls	Exosomal RNA analysis	NA	Diverse RNA types (mRNAs, miRNAs, lincRNAs, tRNAs, piRNAs) were identified in exosomes; exosome RNA profiling could potentially differentiate among PDACs, its precursors (e.g., IPMN), and non-neoplastic conditions
Strijker, 2020 [108]	Plasma cfDNA Patients with metastatic PDACs	Panel including KRAS, GNAS, TP53, SMAD4, CDKN2A, PIK3CA, BRAF, and NRAS	Before therapy (baseline) mostly; during follow-up in 10 patients (1–6 samples per patient)	KRAS and TP53 mutations were the most common ones found; ctDNA was most often found in patients with large tumors and liver metastases, yet in no case with lymph node metastasis only; ctDNA quantity was associated with tumor 3D volume (as measured by imaging), whereas both of them predicted OS
Mohan, 2019 [109]	Plasma cfDNA Patients with locally advanced or metastatic PDAC	WGS and targeted (641 gene panel)	Before therapy	ctDNA was detected more commonly in the metastatic than the locally advanced PDAC cases (87% vs. 62.5%); presence of KRAS copy number gains and mutations were associated with poor prognosis
Liu, 2019 [110]	Plasma cfDNA Patients with pancreatic cancer or IPMN	62 gene panel	NA	Mutations were found in 88% of the patients tested (most common in the TP53, KRAS, CDKN2A, and SMAD4 genes), whereas potentially actionable mutations were also identified (e.g., BRAF, ERBB2); the use of single-strand library preparation enriched the short cfDNA fragments harboring mutations, improving the diagnostic NGS performance concerning early stage pancreatic cancers; short fragment enrichment enhanced the diagnostic capacity of plasma NGS and results were concordant to tissue NGS analysis and the publicly available tissue-based sequencing data
Li, 2019 [111]	Plasma EV-derived RNA PDAC patients and healthy controls	WTS	NA	circRNA profiling from EVs differed between PDACs and healthy controls
Patel, 2019 [112]	CfDNA Patients with resectable or advanced PDAC	54–73 gene panel	During the advanced setting, before or after surgery	TP53 and KRAS mutations were the most common ones found, whereas potentially actionable mutations were also identified in most advanced PDACs; advanced PDACs also showed higher number of aberrations and ctDNA amount (% ctDNA) than the resectable ones; concordance between plasma and tissue NGS was 61% and 52% for TP53 and KRAS mutations, respectively; increased total % ctDNA was associated with shorter OS
Wei, 2019 [113]	Plasma cfDNA Patients with stage III or IV PDAC	560 gene panel	Before (baseline) and during therapy; serial sampling (monitoring) in 17 patients	ctDNA was detected in most patients; compared with stage III, stage IV PDACs showed higher ctDNA quantity; patients with multiple metastatic foci also had higher ctDNA quantity than the ones with fewer foci, reflecting increased tumor burden; in the serial samples, ctDNA quantity was reduced in 11/12 patients who responded to chemotherapy, whereas it was increased in five patients that showed resistance to therapy and progression
Peters, 2018 [114]	Plasma cfDNA Patients with metastatic PDAC	KRAS (exon 2)	At each session, before therapy starts (in total, 1–8 samples per patient)	KRAS mutations were identified in five patients; detection of KRAS mutations in the plasma was associated with serum CA19-9 levels and shorter survival
Riviere, 2018 [115]	Plasma cfDNA Patients with unresectable PDAC or PanNET	68 gene panel	NA	In a cohort composed of gastrointestinal cancers (e.g., colorectal, liver, pancreas), at least one aberration was detected in most patients (most common ones: TP53, KRAS, and PIK3CA), whereas several were potentially actionable; high concordance between liquid and tissue biopsy for four aberrations (KRAS, MYC, and EGFR amplifications; KRAS G12V mutation) was detected
Park, 2018 [116]	Plasma cfDNA PDAC patients	83 gene panel	Before and during therapy	ctDNA was found at most baseline cases (15/17 samples);ctDNA levels were successful to monitor tumor burden, response to therapy or disease progression; the lowest ctDNA levels were found in complete/partial disease response
Berger, 2018 [117]	Plasma cfDNA Patients with metastatic PDAC	7 gene panel	Before therapy (baseline), during the 1st, 2nd, and 3rd line of therapy, and during progression	KRAS and TP53 mutations were the most common ones detected at baseline and during treatment, whereas the mutational landscape was often altered from baseline to the 1st, 2nd, and 3rd lines of treatment; ctDNA quantity dropped from the baseline levels during therapy, whereas it surged during progression; in treatment-naive patients, decrease in ctDNA quantity during therapy was associated with longer PFS
Pishvaian, 2017 [118]	cfDNA and DNA from CTCs Patients with locally advanced and metastatic PDAC	68 gene panel (cfDNA); 50 gene panel (CTCs)	Within 6 weeks from tumor biopsy for most patients	Blood-based liquid biopsy exhibited low concordance compared with the tissue-based molecular analysis, as KRAS mutations were detected in 29% of the liquid, albeit 87% of tissue biopsies; the presence of ctDNA was associated with shorter OS
Vietsch, 2017 [119]	Plasma cfDNA Patients with resectable PDAC	56 gene panel	Before surgery and at disease progression	Although not detecting all mutations found in the tissue-based NGS, liquid biopsy identified a much higher number of alterations not detected in its paired biopsies, reflecting more efficiently the intratumoral heterogeneity; cfDNA collected during progression revealed additional mutations not identified at the pre-operative cfDNA samples
Pietrasz, 2017 [120]	Plasma cfDNA Patients with resectable, locally advanced, or metastatic PDAC	22 gene panel	Before the first cycle of chemotherapy (after surgery for the resectable patients); serial sampling for 8 patients	KRAS, TP53, and SMAD4 mutations were the most common ones detected; the presence of ctDNA was associated with tumor grade and stage (higher detection rates in high-grade and metastatic PDACs); ctDNA presence and quantity was associated with shorter OS in advanced PDACs, whereas its absence conferred longer OS and DFS in resected PDACs
Adamo, 2017 [121]	Plasma cfDNA Patients with PDAC or CP, and healthy controls	50 gene panel	Before therapy	PDACs exhibited higher cfDNA yields than CPs and controls; KRAS mutations were the most common ones detected and were associated with poor prognosis; when both plasma and tissue biopsy were available, plasma NGS failed to detect any mutations detected in their paired tissue biopsies
Chen, 2017 [122]	Plasma cfDNA Patients with stage III or IV PDAC	KRAS (exon 2)	Before (baseline) and during chemotherapy, also with each CT	ctDNA was found in 93.7% of the patients at baseline, even in cases where CA19-9 was undetectable; the combination of ctDNA and CA19-9 increased sensitivity; ctDNA quantity was higher in stage IV than III PDACs, whereas higher ctDNA amount was associated with disease progression and shorter TTP and OS at baseline, being a more significant prognostic marker than serum CA19-9; ctDNA quantity changes at the longitudinal plasma samples predicted response to therapy in most patients
Takai, 2016 [123]	Plasma cfDNA PDAC patients	60 gene panel	Before therapy	At least one mutation was found in all patients; potentially actionable alterations were detected in 14/48 patients (e.g., in ALK, ATM, EGFR, and PIK3CA)
Le Calvez-Kelm, 2016 [124]	Plasma cfDNA Patients with PDAC or CP and healthy controls	KRAS (exons 2 and 3)	NA	Sensitivity was low, as mutations were detected only in 21.1% of the cases; KRAS mutations were more often detected in advanced PDACs, whereas they were also found (at low MAFs though) in a small portion of CPs and healthy controls
San Lucas, 2016 [125]	Exosomal DNA and RNA Patients with PDAC or ampullary carcinoma	WGS WES WTS	Before therapy or during progression	Genomic and transcriptomic profiling was comprehensively performed using exosomal DNA and RNA; potentially actionable alterations (e.g., ERBB2 amplification, NOTCH1 and BRCA2 mutation) were also identified
Ko, 2016 [126]	Plasma cfDNA Patients with locally advanced or metastatic PDAC	54 gene panel	Before (baseline) and during therapy	In this phase II clinical trial, ctDNA was detected in most patients, whereas mutations in KRAS, TP53, ATM, and CDKN2A were the most common ones found at baseline; when paired plasma and tissue biopsy were available in the same patient KRAS mutation detection was 100% concordant between them; most mutations detected at baseline were also found at the follow-up samples, whereas relative ctDNA quantity was linked with the serum CA19-9 levels and tumor burden
Zill, 2015 [127]	Plasma cfDNA Patients with advanced PDAC or biliary carcinoma	54 gene panel	Baseline; serial sampling for 8 patients (monitoring)	Plasma NGS exhibited high sensitivity, specificity, and diagnostic accuracy, whereas it even detected additional alterations from its paired tissue-based NGS; KRAS and TP53 mutations were the most common ones found, whereas actionable alterations (e.g., BRAF or EGFR mutations) were also identified; in the serial samples, changes in ctDNA quantity correlated with the tumor marker (e.g., CA19-9) altered levels, reflecting disease progression or therapy response

Abbreviations: cfDNA, cell-free DNA; ctDNA, circulating tumor DNA; CTCs, circulating tumor cells; cfRNA, cell-free RNA; PDAC, pancreatic adenocarcinoma; pCR, pathologic complete response; NAT, neoadjuvant chemotherapy; PanNET, pancreatic neuroendocrine tumor; EV, extracellular vesicle; NGS, next-generation sequencing; scNGS, single-cell NGS; WGS, whole genome sequencing; WTS, whole transcriptome sequencing; WES, whole exome sequencing; qPCR, quantitative PCR; IPMN, intraductal papillary mucinous neoplasm; CP, chronic pancreatitis; OS, overall survival; RFS, recurrence-free survival; PFS, progression-free survival; DFS, disease-free survival; ORR, overall response rate; PARPi, PARP inhibitor; ICI, immune checkpoint inhibitor; CNAs, copy number alterations; MAFs, mutant allele frequencies; lincRNA, long non-coding RNA; piRNA, piwi-interacting RNA; circRNA, circular RNA; TTP, time to progression.
